# Supplementary figures and images for: Isoginkgetin and Madrasin are poor splicing inhibitors
Source: PLoS One. 2024 Oct 21;19(10):e0310519. doi: 10.1371/journal.pone.0310519 (PMC11493277; doi:10.1371/journal.pone.0310519)

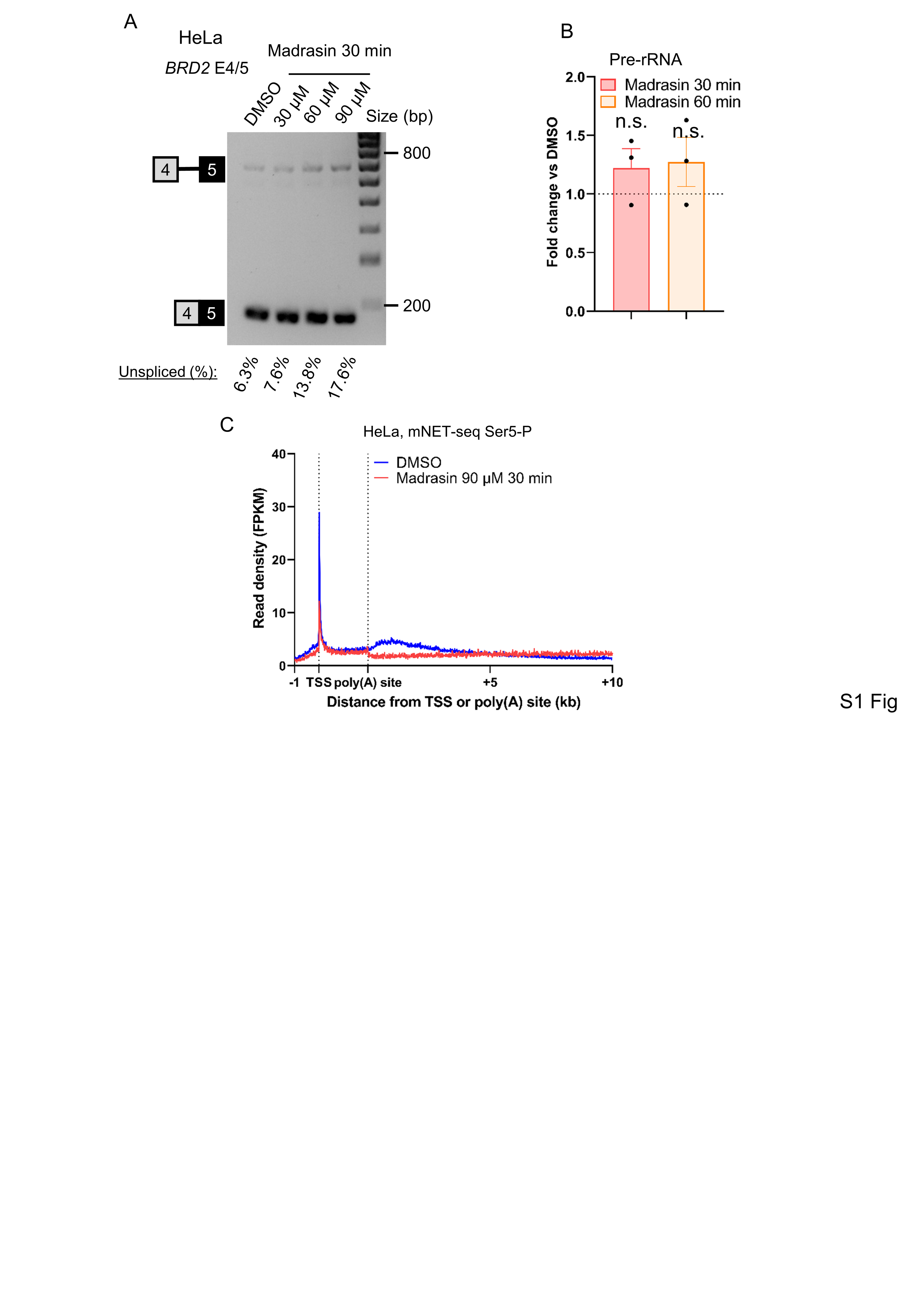

Supplement: S1 Fig — (A) RT-PCR with primers amplifying the intron located between exons 4 and 5 of BRD2. HeLa cells were treated with DMSO or 30 μM, 60 μM, or 90 μM Madrasin for 30 min. The location of the spliced and unspliced RNA is shown on the left of the panel. The percentages of unspliced RNA compared to total (spliced + unspliced) are shown below. (B) qRT-PCR with primers amplifying a region from the pol I transcribed pre-rRNA. HeLa cells were treated with DMSO or 90 μM Madrasin for 30 min (red) or 60 min (orange). cDNA was generated with random hexamers. Values are normalised to the 7SK snRNA and shown as relative to DMSO, mean ± SEM, n = 3 biological replicates. Statistical test: two-tailed unpaired t-test. P-value: n.s. not significant. (C) Metagene profile of pol II CTD Ser5-P mNET-seq performed in HeLa cells treated with DMSO (blue) or 90 μM Madrasin for 30 min (red) on scaled expressed protein-coding genes. (TIF) [file pone.0310519.s001.tif]

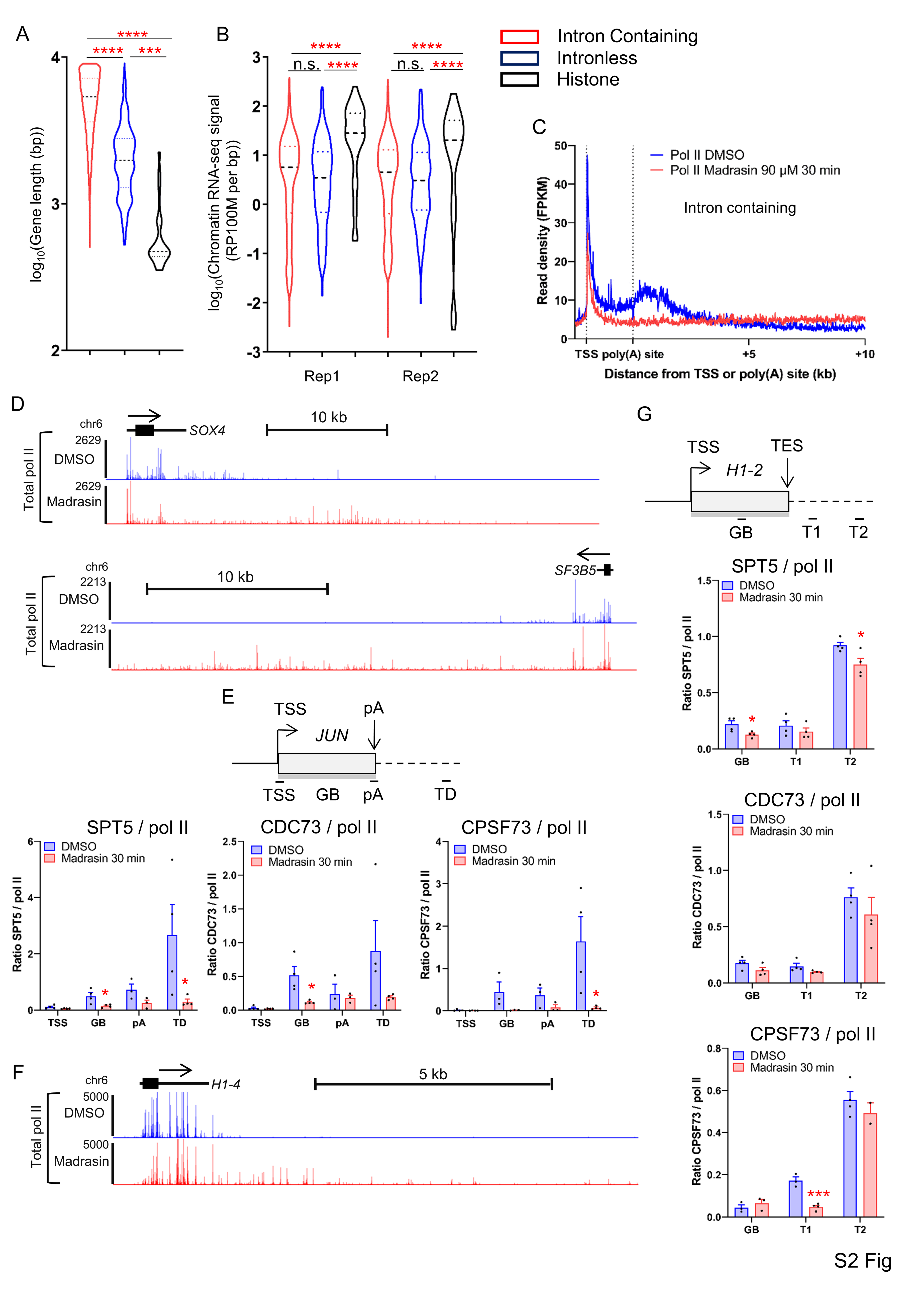

Supplement: S2 Fig — (A) Violin plots, defined as min to max values, first and third quartiles (thin dotted lines), and mean (thicker dotted line), showing the log10 of gene length distribution (in bp) for intron-containing (red), intronless (blue), and histone (black) genes. Statistical test: Wilcoxon rank sum test. P-value: *** < 0.001, **** < 0.0001. (B) Violin plots, defined as min to max values, first and third quartiles (thin dotted lines), and mean (thicker dotted line), showing the log10 of gene expression distribution, from two biological HeLa chromatin RNA-seq experiments, for intron-containing (red), intronless (blue), and histone (black) genes. Statistical test: Wilcoxon rank sum test. P-value: ns: not significant, **** < 0.0001. (C) Metagene profile of total pol II mNET-seq performed in HeLa cells treated with DMSO (blue) or 90 μM Madrasin for 30 min (red) on scaled expressed intron-containing genes. (D) Screenshots of the genome browser total pol II mNET-seq DMSO (blue) and Madrasin (red) tracks of the intronless genes SOX4 and SF3B5. The arrow indicates the sense of transcription. (E) Ratios of SPT5 / total pol II, CDC73 / total pol II, or CPSF73 / total pol II from ChIP-qPCR on the intronless gene JUN in HeLa cells treated with DMSO (blue) or 90 μM Madrasin for 30 min (red). Mean ± SEM, n = 3 biological replicates. Statistical test: two-tailed unpaired t-test. P-value: * < 0.05. (F) Screenshot of the genome browser total pol II mNET-seq DMSO (blue) and Madrasin (red) tracks of the histone gene H1-4. The arrow indicates the sense of transcription. (G) Ratios of SPT5 / total pol II, CDC73 / total pol II, or CPSF73 / total pol II from ChIP-qPCR on the histone gene H1-2 in HeLa cells treated with DMSO (blue) or 90 μM Madrasin for 30 min (red). Mean ± SEM, n = 3 biological replicates. Statistical test: two-tailed unpaired t-test. P-value: * < 0.05, *** < 0.001. (TIF) [file pone.0310519.s002.tif]

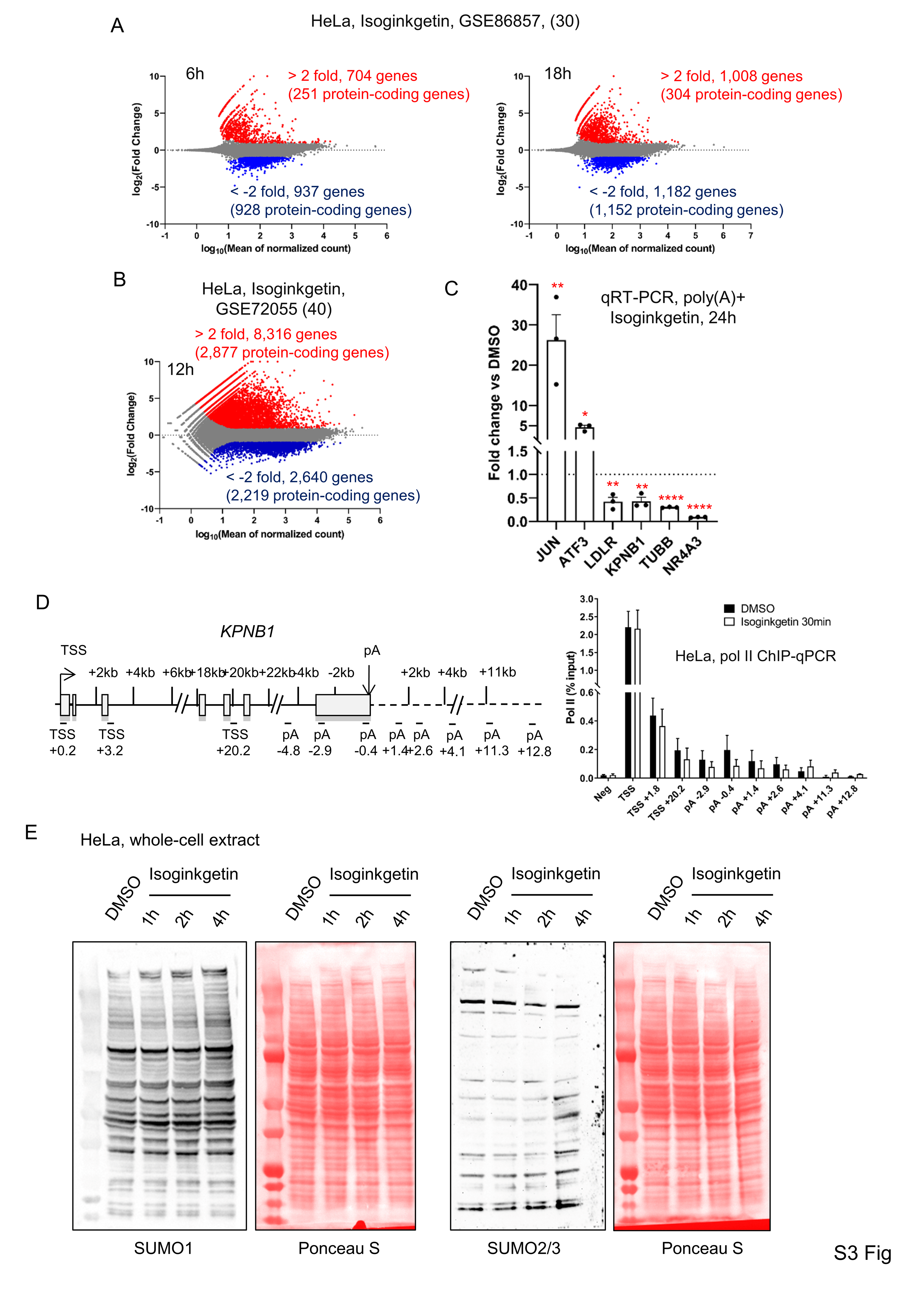

Supplement: S3 Fig — (A) MA plot of total RNA-seq in HeLa cells treated with DMSO or Isoginkgetin for 6h or 18h. Significantly upregulated genes are shown in red while significantly downregulated genes are shown in blue. Other genes are shown in grey. The numbers of differentially expressed genes are shown on the figure. (B) MA plot of total RNA-seq in HeLa cells treated with DMSO or Isoginkgetin for 12h. Significantly upregulated genes are shown in red while significantly downregulated genes are shown in blue. Other genes are shown in grey. The numbers of differentially expressed genes are shown on the figure. (C) qRT-PCR with primers amplifying different protein-coding genes. HeLa cells were treated with DMSO or 30 μM Isoginkgetin for 24h. cDNA was generated with oligo(dT). Values are normalised to the protein-coding gene GAPDH and shown as relative to DMSO, mean ± SEM, n = 3 biological replicates. Statistical test: two-tailed unpaired t-test. P-value: * < 0.05, ** < 0.01, **** < 0.0001. (D) Total pol II ChIP-qPCR of the intron-containing gene KPNB1 in HeLa cells treated with DMSO (black) or 30 μM Isoginkgetin (white) for 30 min. Mean ± SEM, n = 3 biological replicates. Statistical test: two-tailed unpaired t-test. P-value: not significant. (E) Western blot on whole cell extract of HeLa cells treated with DMSO or 30 μM Isoginkgetin for 1h, 2h, or 4h. The antibodies are indicated at the bottom, with Ponceau S staining used as loading control. (TIF) [file pone.0310519.s003.tif]

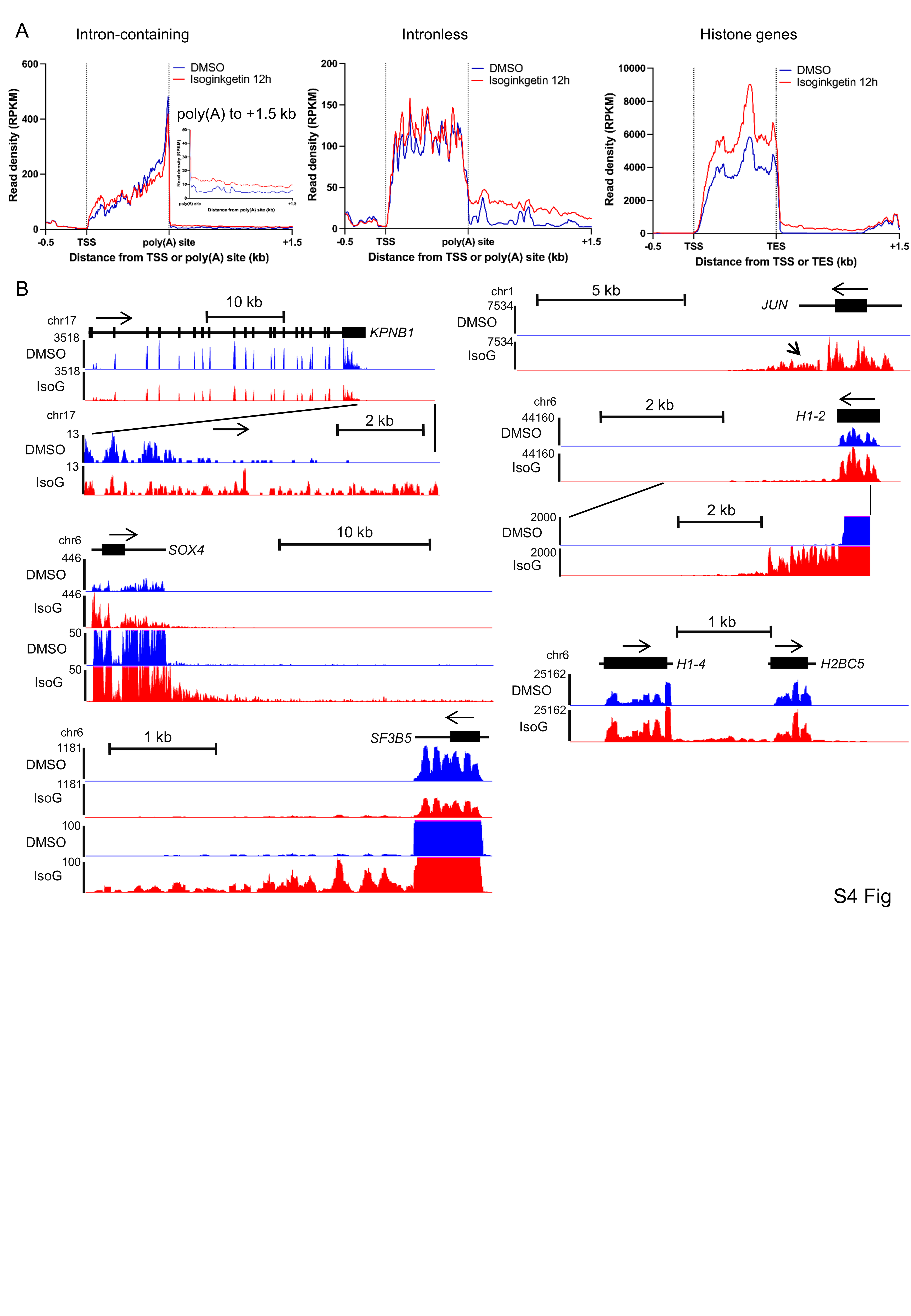

Supplement: S4 Fig — (A) Metagene profile of total RNA-seq performed in HeLa cells treated with DMSO (blue) or Isoginkgetin (red) for 12h on scaled expressed intron-containing, intronless, and histone genes. The metagene profiles are shown as the average of two biological replicates. (B) Screenshots of the genome browser tracks for HeLa RNA-seq treated with DMSO (blue) or Isoginkgetin (red) for 12h on the intron-containing gene KPNB1, intronless genes SOX4, SF3B5, and JUN, and histone genes H1-2, H1-4, and H2BC5. The arrow indicates the sense of transcription. (TIF) [file pone.0310519.s004.tif]
