# Supplementary material for: Isoginkgetin and Madrasin are poor splicing inhibitors
Source: PLoS One. 2024 Oct 21;19(10):e0310519. doi: 10.1371/journal.pone.0310519 (PMC11493277; doi:10.1371/journal.pone.0310519)
Supplement: Attachment — Submitted filename: Response_To_Reviewers_V2.docx [file pone.0310519.s005.docx]

Reviewer #1: 1. It would be great to show some of the qPCR data using the ddPCR method to understand the exact defect in the splicing or at the transcriptional level.

We thank the reviewer for their positive comments. While interesting, we think that ddPCR is beyond the scope of the study as the main message of the manuscript is that Isoginkgetin and Madrasin are poor splicing inhibitors and should therefore not be used to inhibit pre-mRNA splicing.

2. How are RNA Pol II subunit expression levels altered following Madrasin treatment compared to Isoginkgetin? Also, as a positive control, some of the figures in Figure 3 (such as 3F) are better suited for a PladB treatment comparison. 3. Similarly, for short-term treatment of Isoginkgetin, PladB comparison will be helpful to justify that splicing is secondary to transcriptional inhibition.

We added to Fig 1E western blots of the large subunit RNA polymerase II (Rpb1), SF3B1, α-tubulin, and histone H3 performed on chromatin/nucleoplasm/cytoplasm fractions of HeLa cells treated for 1 hour with DMSO, Isoginkgetin, Madrasin, Pladienolide B, and Herboxidiene. While SF3B1 (as positive control) is decreased on chromatin and increased in nucleoplasm and cytoplasm fractions after treatment with PlaB, HB, and Madrasin, Isoginkgetin has no effect on SF3B1. For total Rpb1, none of the inhibitors has an effect on its cellular distribution indicating that the decrease in transcription is not due to degradation of pol II.

For the comparison of Isoginkgetin and Madrasin to PlaB, we have extended the Discussion to describe the results we obtained on the effect of PlaB on splicing and transcription and how they differ to what we observe for Isoginkgetin and Madrasin.

Added to the Discussion- “The effects of Isoginkgetin and Madrasin on transcription and pre-mRNA splicing also differ from the effects of SF3B1 inhibitors as we and others have found that PlaB rapidly inhibits co-transcriptional splicing (30 min) and reduces P-TEFb recruitment to chromatin, resulting in an increased pol II pausing, reduced transcription elongation, and increased premature transcription termination (5, 7, 19-21). These results indicate that Isoginkgetin and Madrasin are unlikely to be SF3B1 inhibitors.”

Reviewer #2: In this study, authors have investigated the effect of Madrasin and Isoginkgetin, two non-SF3B1 splicing inhibitors, on splicing and transcription. The results show that Madrasin and Isoginkgetin are poor splicing inhibitors using assays like RT-PCR for exons 2 and 3 of DNAJB1 and exons 4 and 5 of BRD2, nascent transcription activity via 5’EU incorporation, metagene profiling of total pol II mNET-seq and qRT-PCR with primers amplifying different protein-coding genes. My recommendation is to accept the manuscript in current without any additional changes.

We thank the reviewer for their positive comments.
